# A randomized controlled pilot study investigating adherence to blood pressure diaries with personal pictures in stroke follow-up care

**DOI:** 10.1007/s00508-025-02530-w

**Published:** 2025-04-22

**Authors:** Clemens V. Farr, Johanna Ebner, Marie Beatrice Lang, Sonja Zehetmayer, Katharina Trawnicek, Stefan Greisenegger, Wolfgang Serles, Thomas Berger, Patrick Altmann

**Affiliations:** 1 Department of Neurology, Medical University of Vienna, Waehringer Guertel 18-20, 1090 Vienna, Austria; 2 Comprehensive Center of Clinical Neurosciences and Mental Health, Medical University of Vienna, Vienna, Austria; 3 Center for Medical Data Science, Medical University of Vienna, Vienna, Austria

**Keywords:** Blood pressure documentation, Compliance, Cerebrovascular, Prevention, Transient ischemic attack

## Abstract

**Introduction:**

Blood pressure (BP) management is essential in secondary stroke prevention. Strategies to ensure continuous home BP monitoring are needed. Few studies investigated factors influencing adherence to home BP management. Therefore, we designed a pilot study to investigate the feasibility of keeping BP diaries (BPDs) with personal images.

**Methods:**

In this prospective trial, we randomized persons with a diagnosis of stroke or transient ischemic attack into two groups: (i) 10 patients received a personalized BPD with pictures of their choosing and (ii) 10 patients received a BPD without photographs. We instructed participants in both groups to document their BP at home twice daily over 28 days. Adherence was defined as the number of BP measurements performed relative to the maximum number of recommended measurements. We assessed patient reported outcomes as exploratory endpoints.

**Results:**

We found no statistically significant difference in mean adherence between the control group (64%) and the intervention group (69%). The BP was within the recommended range and precision of documentation was high in both groups, without statistically significant differences. Patient reported outcomes such as depression scores did not differ significantly between study groups.

**Conclusion:**

Our findings underline the relevance to investigate aspects of adherence to home BP management suggesting the inclusion of patient-provided pictures to be feasible.

**Supplementary Information:**

The online version of this article (10.1007/s00508-025-02530-w) contains supplementary material, which is available to authorized users.

## Introduction

Stroke is the second leading cause of death worldwide and a major cause of permanent disability [1]. The increasing incidence of stroke and resulting death or disability is partly attributable to modifiable lifestyle factors [2]. Therefore, secondary stroke prevention is an integral part of stroke management. Arterial hypertension is a key modifiable risk factor in stroke prevention. Blood pressure (BP) lowering yields a 25–30% risk reduction for stroke recurrence [3, 4]. High BP is the most significant contributor to disability-adjusted life years in stroke [2].

Accurate BP measurement is a prerequisite for an effective antihypertensive therapy and BP monitoring is recommended twice daily [5]. Due to the impracticality of longitudinal clinic-based BP measurements, home monitoring of BP recorded in blood pressure diaries (BPDs) has gained popularity [6]. The affordability, convenience and patient empowerment along with its potentially higher prognostic value have driven its adoption. [7]. The BP home monitoring is feasible in people with poor BP control and the impact on secondary prevention is well-documented [8, 9]. Home-monitored BP reduces arterial BP, regardless of changes in antihypertensive therapy and had more favorable cardiovascular outcomes than BP measured in the hospital [8, 10].

However, fostering a routine for regular home BP measurements remains challenging. Studies on the adherence of patients to BP home measurements are scarce, which may obscure the potential to optimize secondary stroke prevention. Moreover, the lack of data precludes sample size calculations for studies investigating ways of boosting adherence to BPDs (i.e., improving the prognostic accuracy of recorded BP values). Addressing these shortcomings, we designed a randomized controlled pilot study to investigate whether the proper maintenance of BPDs is enhanced by the inclusion of pictures individually selected by patients for their personally meaningful and positive emotional content.

## Methods

### Trial design

This randomized controlled single-blinded trial was designed as a single-center pilot study. It was conducted in accordance with the guidelines for pilot studies of the ethics review board at the Medical University of Vienna. For the study 20 patients were recruited at the Department of Neurology of the Medical University of Vienna and randomized 1:1 into two groups. We used the BPD templates of the American Heart Association as a model [11]. Accordingly, BPDs consisted of tables including columns for date, time and BP (1 page per week) printed on the right-hand pages of an A5 booklet. Participants in the intervention group (picture-BPD; P‑BPD) received BPDs which featured patient-chosen pictures, provided over a secure online connection, on the left-hand pages. Patients in the control group obtained regular BPDs (R-BPD) without pictures. Two authors with access to the randomizing software, handled patient recruitment and group assignment. According to current guidelines, we instructed both groups to monitor BP twice daily for 28 days after discharge [5]. A follow-up visit occurred after this study period, where patients handed in their BPDs and completed patient-reported outcome questionnaires. Data were curated by an investigator blinded to group allocation. Blinding was performed by covering the left-hand pages of BPDs and pseudonymization.

### Participants

We recruited patients diagnosed with transient ischemic attack (TIA) or stroke (ischemic or hemorrhagic) upon admission. Study inclusion was irrespective of sociodemographic factors. Exclusion criteria were a modified Rankin Scale score of ≥ 4, inability to provide informed consent and legal guardianship. No protocol changes occurred after the inclusion of the first participant.

### Outcomes

The primary endpoint was adherence to BPDs defined as the number of complete BP measurements (i.e. entries stating date, time and systolic as well as diastolic BP, maximum of two per day) divided by the maximum possible measurements (56). The number of complete entries was extracted from the returned BPD. We considered a one entry difference between groups clinically meaningful, as each additional measurement may aid in treatment adjustments and uncovering daily BP variability. Another primary endpoint was the distribution and variability of the number of complete BP measurements, aiding sample size calculations for future studies.

Secondary endpoints were additional dimensions of BPD feasibility: to identify potential effects of personalized BPDs on BP we calculated the mean systolic and diastolic BP for each patient using all of their respective BPD entries. We then computed the overall mean of these individual patient means for each group, enabling us to compare the average BP between study groups. The proportion of documented values ending with “0” or “5” served to assess the extent of rounding (i.e. the precision of documentation) [12]. Health-related quality of life and possible implications of picture-based BPDs were investigated using patient-reported outcome (PRO) questionnaires (Patient Global Impression of Change Scale [PGIC], 36-Item Short Form Health Survey [SF-36] and Hospital Anxiety and Depression Scale [HADS], [13–15]). These outcomes were included to explore whether personalized diaries influence mental well-being, as increased engagement and a sense of empowerment can positively impact mood and anxiety levels.

Exploratory endpoints examined patients’ perspectives on BPDs through a survey (13 questions in each group). Likert-type scale answers were pooled in a way to separate favorable or neutral from opposing ratings.

## Statistics

We report categorical variables as absolute frequencies and percentages and continuous variables as median ± interquartile range (IQR) or as mean ± standard deviation (SD) as appropriate. As a pilot study, our study did not include a priori hypotheses or a sample size calculation. Statistical tests were performed to check for underlying distributions and patient characteristic balance. Categorial variables were tested with the χ^2^-test or (for cases of expected absolute frequencies < 5) Fisher’s exact test. We tested continuous variables for normal distribution by the Shapiro-Wilk test. Between-group differences in continuous variables were tested with an unpaired two-sample two-tailed t‑test or Mann-Whitney U test as appropriate. Variances were tested with an F‑test. A *p*-value of < 0.05 was considered significant. There were no multiple comparisons. We performed randomization with an online tool (www.randomizer.at) [16]. In cases of missing values for PROs, we report the fraction of missing values and, where applicable, applied the personal mean score imputation method for completion [17]. Statistical analysis was performed using SPSS 21.0 (SPSS Inc, Chicago, IL, USA).

### Data security

Our study protocol adhered to current data protection guidelines set by the country of Austria.

## Results

### Patient and disease characteristics

**Fig. 1:**
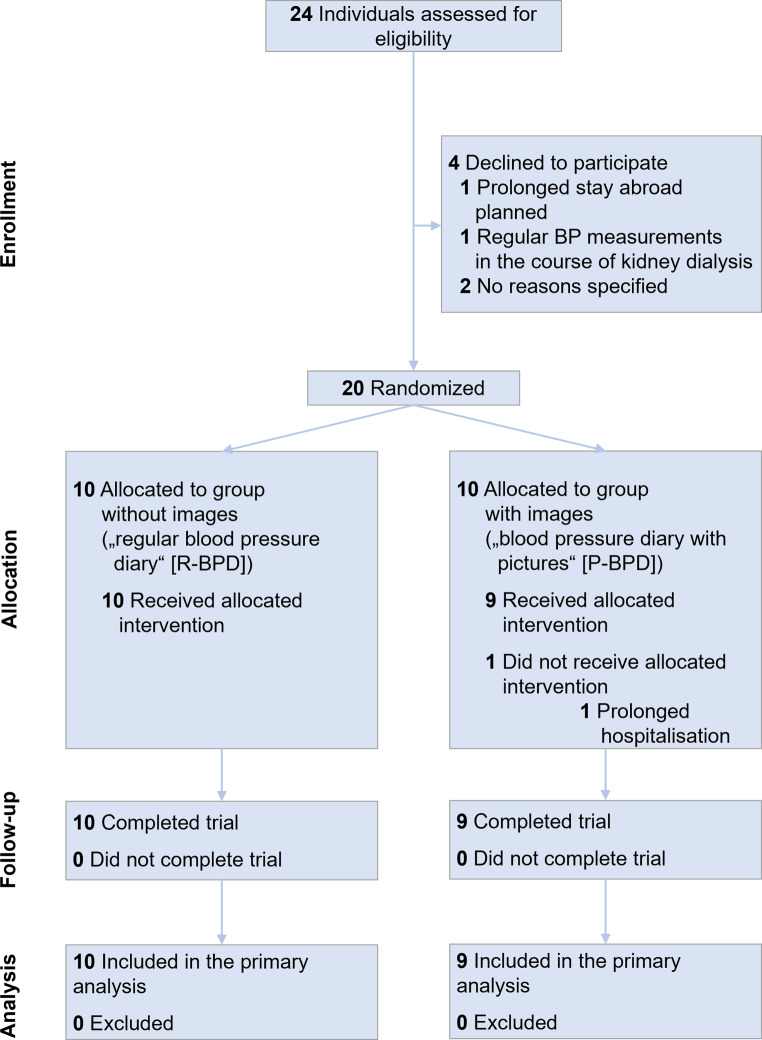
CONSORT flow diagram [30]. The diagram illustrates patient recruitment, randomization and follow-up in this study. *BP* blood pressure

Figure 1 outlines the participants’ progression through the study. Recruitment ran from July 2023 (first patient in) and lasted until October 2023 (last patient in). We screened 24 patients for eligibility and 4 declined participation for the following reasons: (i) planning a prolonged vacation (*n* = 1), (ii) feeling that BP is sufficiently monitored during routine outpatient care (*n* = 1) and (iii) not being interested (*n* = 2). One patient dropped out shortly after randomization due to unexpected prolonged hospitalization. Study visits lasted from August 2023 (first patient out) through October 2023 (last patient out). Final analysis ended in October 2023.

Table 1 shows sociodemographic and clinical information of study completers. The mean age was 61 ± 16 (SD) years. Most participant characteristics, including the presence of arterial hypertension and other comorbidities, were distributed evenly between both groups (see also Supplementary Table 1). A difference was found in the distribution of gender (*p* = 0.023). As this was a randomized study with a small sample size, we cannot reasonably relate this to other study outcomes.

**Table 1:** Sociodemographic and clinical information of participants completing this study

Parameter	Category	Total cohort	R‑BPD	P‑BPD
		*n* = 19	*n* = 10	*n* = 9
Age^a^	Years	61 ± 16	61 ± 11	62 ± 21
Gender^b^	Female	9 (47)	2 (20)	7 (78)
	Male	10 (53)	8 (80)	2 (22)
Housing before study^b^	Home self-reliant	10 (53)	5 (50)	5 (56)
	Home with assistance (family/friends)	8 (42)	4 (40)	4 (44)
	Home with assistance (professional)	1 (5.)	1 (10)	0 (0)
	Nursing home	0 (0)	0 (0)	0 (0)
	Retirement home	0 (0)	0 (0)	0 (0)
Housing after discharge^b^	Home self-reliant	7 (37)	3 (30)	4 (44)
	Home with assistance	9 (47)	4 (40)	5 (44)
	Nursing home	2 (11)	2 (20)	0 (0)
	Rehabilitation center	1 (5)	1 (10)	0 (0)
Neurovascular diagnosis^b^	Ischemic stroke	15 (79)	9 (90)	6 (67)
	Hemorrhagic stroke	1 (5)	0 (0)	1 (11)
	TIA	3 (16)	1 (10)	2 (22)
MRS (at first clinical assessment upon arrival at the hospital, i.e. before study inclusion)^b^	0–1	12 (63)	5 (50)	7 (78)
	2–3	5 (26)	3 (30)	2 (22)
	4–5	2 (11)	2 (20)	0 (0)
MRS (at discharge from the hospital, i.e. after study inclusion)^b^	0–1	14 (74)	6 (60)	8 (89)
	2–3	5 (26)	4 (40)	1 (11)
	4–5	0 (0)	0 (0)	0 (0)
MRS (at study visit following 28 days of BPD usage)^b^	0–1	15 (79)	7 (70)	8 (89)
	2–3	4 (21)	3 (30)	1 (11)
	4–5	0 (0)	0 (0)	0 (0)
Antihypertensives (at discharge)^b^	Yes	12 (63)	7 (70)	5 (56)
	No	7 (37)	3 (30)	4 (44)
Arterial hypertension^b^	Yes	11 (58)	7 (70)	4 (44)
	No	8 (42)	3 (30)	5 (56)
Comorbidities leading to recommendation of BPD before study^b^	Yes	11 (58)	6 (60)	5 (56)
	No	8 (42)	4 (10)	4 (44)
Patient-reported use of BPD before study^b^	Yes	10 (53)	5 (50)	5 (56)
	No	9 (47)	5 (50)	4 (44)
Patient-reported use of symptom tracker(s) before study^b^	Yes	2 (11)	1 (10)	1 (11)
	No	17 (90)	9 (90)	8 (89)

*BPD* blood pressure diary; *MRS* modified Rankin Scale (on admission); *P‑BPD* picture blood pressure diary; *R‑BPD* regular blood pressure diary; *TIA* transient ischemic attack

^a^mean ± standard deviation

^b^absolute number (percentage)

### Adherence to BPD

**Fig. 2:**
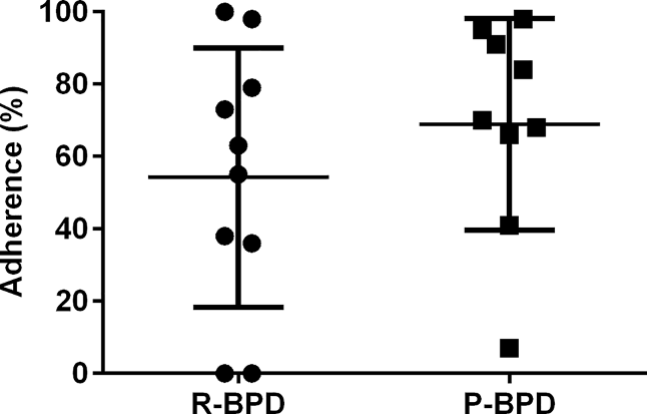
Adherence to BPDs in our study groups. Points (R-BPD) and squares (P-BPD) represent adherence of our individual participants as defined by the proportion of complete entries relative to the recommended number of measurements. The horizontal black lines represent the group’s overall mean adherence. Error bars represent the standard deviation*. P‑BPD* picture blood pressure diary; *R‑BPD* regular blood pressure diary

Mean (SD) adherence in the total cohort was 66% (30%), 64% (33%) in our control group and 69% (29%) in our P‑BPD group (Fig. 2). This equates to an average of 3 more entries in our intervention group. This difference was not statistically significant. Complete entries were normally distributed and the standard deviations of both groups were not significantly different.

### Levels and precision of documented BP and depression scores

Table 2 lists mean BP values in our cohort. There was no statistical difference in mean BP between our groups. The proportions of BP values ending on “0” or “5” ranged from 11% to 22% and did not statistically differ between groups. Our primary interest concerning patient-reported outcome measures was mood (Table 3): on average, participants with P‑BPD scored 3 points lower in the HADS depression scale. This was not statistically significant. Two participants declined to complete questionnaires to explore PROs, without explanation.

**Table 2:** BP values documented by participants

Parameter		Total cohort	R‑BPD	P‑BPD
		*n* = 19	*n* = 10	*n* = 9
Systolic BP (mm Hg)^a^		124 ± 12	126 ± 11	123 ± 13
Diastolic BP (mm Hg)^a^		72 ± 14	75 ± 17	72 ± 13
Proportion of BP values ending on “0” or “5” (%)^a^	Systolic	22 ± 8	21 ± 11	22 ± 7
	Diastolic	17 ± 17	21 ± 21	11 ± 17

*BP* blood pressure; *P‑BPD* picture blood pressure diary; *R‑BPD* regular blood pressure diary

^a^mean ± standard deviation

**Table 3:** Patient-reported outcome measures

Survey	Domains	R‑BPD	P‑BPD
HADS^a^	Depression (points)	7 ± 4	4 ± 4
	Anxiety (points)	5 ± 3	7 ± 4
PGIC^b^	Considerably better	3 (33)	2 (25)
	Better	0 (0)	2 (25)
	Moderately better	0 (0)	1 (13)
	Somewhat better	1 (11)	1 (13)
	a little better	2 (22)	0 (0)
	Almost no change	1 (11)	2 (25)
	No change	2 (22)	0 (0)
SF-36^a^	Physical functioning (%)	63 ± 33	65 ± 35
	Role limitations due to physical health (%)	34 ± 48	31 ± 37
	Role limitations due to emotional problems (%)	46 ± 50	25 ± 39
	Energy/fatigue (%)	39 ± 22	44 ± 23
	Emotional well-being (%)	50 ± 16	57 ± 26
	Social functioning (%)	69 ± 18	66 ± 33
	Pain (%)	78 ± 22	72 ± 37
	General health (%)	43 ± 17	50 ± 27

Completed questionnaires (R-BPD/P-BPD): 9/8 (HADS and PGIC); 7/7 (SF-36). Incomplete questionnaires (R-BPD/P-BPD): completion through imputation: 2/2 (SF-36); imputation not applicable: 1/0 (SF-36)

*HADS* Hospital Anxiety and Depression Scale; *P‑BPD* picture blood pressure diary; *PGIC* Patients’ Global Impression of Change Scale; *R‑BPD* regular blood pressure diary; *SF-36* 36-Item Short Form Health Survey

^a^mean ± standard deviation

^b^absolute number (percentage)

### Other PRO measures

On average, participants using P‑BPDs scored 2 points higher in the HADS anxiety scale than participants in the control group, which was not statistically significant. Of the SF-36 questionnaires 5 were incomplete and 4 contained at least 1 answer in each subscale, which enabled personal mean score imputation for completion [17]. We observed a balanced distribution of responses in the total cohort and both groups across all PGIC and most SF-36 categories (Table 3).

### BPD feasibility and user experience

Table 4 lists exploratory outcome parameters on patient engagement with BPDs and two patients declined to complete the questionnaire without explanation. Overall, satisfaction with BPDs was high. In the R‑BPD group 56% felt that inclusion of images into BPDs could increase their adherence and 75% of P‑BPD users reported increased motivation in home monitoring of their BP due to the visuals. Consistently, most participants in both groups preferred picture-personalized BPDs over conventional ones. In most instances, BP was measured and documented by the participants themselves. The high level of autonomy in our cohort was reflected by our participants stating they rarely needed to be reminded of measurements by others. None of the exploratory endpoints differed significantly between our study groups.

**Table 4:** Patient feedback on BPD feasibility and experience

Self-reported experience^a^	Answer	R‑BPD	P‑BPD
Do you believe that you would be more inclined to measure your blood pressure at home if you had a personalized blood pressure diary, perhaps with personal pictures? (yes/no)	Yes	5 (56)	*
	No	4 (44)	*
Do you feel that, due to the inserted images, you were more motivated to measure your blood pressure at home? (yes/no)	Yes	*	6 (75)
	No	*	2 (25)
How likely are you to prefer blood pressure journals with images in the future? (1–5)	Likely [1–2]	5 (56)	4 (50)
	Neutral [3–4]	3 (33)	3 (378)
	Definitely not [5]	1 (11)	1 (13)
How often have you discussed your blood pressure journal with other people? (frequency)	> 6 times	1 (11)	1 (13)
	4–6 times	1 (11)	0 (0)
	2–4 times	2 (22)	4 (50)
	Once	2 (22)	1 (13)
	Never	3 (33)	2 (25)
How frequently have you shared your blood pressure journal with friends, acquaintances, or caregivers? (frequency)	> 6 times	2 (22)	0 (0)
	4–6 times	1 (11)	0 (0)
	2–4 times	1 (11)	4 (50)
	Once	1 (11)	1 (13)
	Never	4 (44)	3 (38)
How satisfied were you overall with the blood pressure diary? (1–5)	Satisfied [1–2]	7 (78)	8 (100)
	Neutral [3–4]	2 (22)	0 (0)
	Dissatisfied [5]	0 (0)	0 (0)
How satisfied were you with the layout of the blood pressure journals? (1–5)	Satisfied [1–2]	8 (89)	6 (75)
	Neutral [3–4]	1 (11)	2 (25)
	Dissatisfied [5]	0 (0)	0 (0)
Were the characters in the blood pressure diary sufficiently legible for you? (yes/no)	Yes	9 (100)	8 (100)
	No	0 (0)	0 (0)
Are you aware of how important it is to measure blood pressure at home after experiencing a stroke or heart attack? (yes/no/partially)	Yes	6 (67)	8 (57)
	No	1 (11)	0 (0)
	Partially	2 (22)	0 (0)
Were you informed upon discharge about how to correctly measure blood pressure? (yes/no/partially)	Yes	7 (78)	6 (75)
	No	2 (22)	1 (13)
	Partially	0 (0)	1 (13)
Who conducted the blood pressure measurements on you during the ongoing study? (me/caregiver/both)	Me	7 (78)	8 (100)
	Caregiver	0 (0)	0 (0)
	Both	2 (22)	0 (0)
Who documented the results of the blood pressure measurements in the blood pressure journal? (me/caregiver/both)	Me	7 (78)	8 (100)
	Caregiver	0 (0)	0 (0)
	Both	2 (22)	0 (0)
Did another person remind you to measure your blood pressure twice daily (e.g., your partner, a family member or a caregiver)? (yes/no)	No	7 (78)	6 (75)
	Yes	2 (22)	2 (25)
Did you fabricate measurements in your blood pressure journal? (yes/no)	No	9 (100)	8 (100)
	Yes	0 (0)	0 (0)

Patients surveyed (R-BPD/P-BPD): 9/8. Answers to Likert scale questions (1–5) are reported as grouped answers weighed positively (1–2), neutral (3–4) or negatively (5)

*P‑BPD* picture blood pressure diary; *R‑BPD* regular blood pressure diary

^a^absolute number (percentage)

*not surveyed in this group

## Discussion

The BP home measurements provide high prognostic accuracy for assessing stroke risk, making BPDs valuable tools in secondary stroke prevention [18, 19]; however, their effectiveness in guiding treatment depends on patient adherence, highlighting the importance of studying factors that influence BPD adherence in people at risk for stroke. Earlier studies focused on other aspects of BPDs (e.g. self-titration of antihypertensives) and used coarse measures of BPD adherence [20–25] or were not conducted in people with neurovascular diagnoses [12, 26]. Moreover, these studies did not assess adherence variability, preventing sample-size calculations for interventional studies. Our pilot study addressed these gaps, by investigating adherence to BPDs with personal, positive images provided by patients as well as patient preferences in their use of BPDs.

At roughly 60%, overall adherence to home BP measurements was suboptimal, emphasizing the need for interventions to motivate people with stroke or TIA to self-monitoring. Considering the Hawthorne effect that an observation itself influences behavior, the mean completion rate may even constitute an overestimation [27]. The integration of personalized visuals into BPDs led to a trend of higher adherence in our intervention group and may therefore hold potential to address low BPD adherence; however, this trend was not statistically significant. Therefore, our study does not provide evidence that personalized visuals improve BPD adherence, although the small sample size may have limited our ability to detect an effect. Earlier studies measured adherence to BPDs by counting time intervals (weeks or days) containing at least one entry but this approach overlooks BP variability within the defined time interval [21–23]. Furthermore, as we consider each additionally available BP value to be clinically meaningful, a mere count of days with at least one entry may prevent identification of arterial hypertension in some patients. By this definition, one study reported a BPD adherence of nearly 100% [22]. Applying the same definition of adherence on our data would lead to greater estimates of adherence (74% ± 29%total cohort, 71% ± 32% R‑BPD and 77% ± 27% P‑BPD). This discrepancy highlights that coarse measures may overestimate adherence. Other adherence metrics in the literature include months with enough recordings for a management decision to be made [24], dichotomization into adhering and non-adherent [25] or categorizing by total number of entries [20].

In addition to health state evaluation and to discern potential impacts on BP, we examined BP values. The BP was within the range recommended for stroke patients in the total cohort and both groups [28]. Consistent with the even distribution of clinical characteristics, BP did not statistically differ between study groups. Imprecise BPD entries may cause misclassification of the severity of arterial hypertension and impact therapeutic decisions [26]. Hence, precision of BP documentation is an important determinant of the efficacy of BPDs [24]. Precision of documentation is measured by the proportion of entries ending in “0” or “5”, with expected proportions of one fifth in the absence of rounding [12]. The proportions observed in our cohort and in both study groups barely exceeded this threshold, with no statistically significant difference detected between study groups. We concluded that participants in both groups accurately documented their BP. The high baseline accuracy likely limited the opportunity to detect further improvements with P‑BPDs.

Individualized interventions empower patients, increasing their sense of control and potentially improving adherence [29]. Building on patient empowerment inherent to BPDs, we implemented personalization in the form of patient-provided images, additionally leveraging the emotional and associative impact of visuals. Consistently, we assessed the intervention’s effect on mental health. Participants with P‑BPD showed a 3-point lower score on the HADS depression scale on average but this difference did not reach statistical significance. We also observed statistically non-significant, mildly increased role limitations due to emotional problems and slightly higher anxiety in the intervention group. Given the small sample size and pilot nature of the study, these seemingly paradoxical findings require further exploration in a follow-up study.

Almost all study participants were satisfied with the use of BPDs and more than half shared their BPD at least once with other people, highlighting the emotional and associative benefits of BPDs; however, the insertion of pictures into BPDs did not increase the frequency of sharing or discussing the BPDs with other people. Those in the P‑BPD group submitted pictures showing family, friends, pets and holiday memories. They reported that the inserted images did increase their motivation to monitor their BP, extending the supportive effects of BPDs. While this did not translate into a significant difference regarding BPD adherence, patients in the control group felt that personalized images would improve their adherence. Most participants in both groups reported that they would prefer P‑BPDs over R‑BPDs in the future. Therefore, we regard our proposition of introducing personalized images to BPD to be well-received in our study.

As we conceived this investigation as a pilot study, we must report significant limitations evolving around our short observation period and small sample size. The rather short duration of our study limited our ability to assess whether adherence is sustained over a longer period. The short study period may explain why mean BP values as assessed in our study were a result of higher adherence to hypertension treatment following hospital discharge. Furthermore, we are unable to determine long-term effects of our method on BPD adherence. A longer observation period may have revealed different aspects of personalized BPDs and their suspected role in greater adherence. Furthermore, the second limitation due to the preliminary nature of our study was a small sample size. This has prevented us from detecting statistical differences and perform subgroup or regression analyses. A greater statistical power may have been helpful to corroborate our findings of certain patient reported outcomes (such as mental health aspects).

Also, age and sex matching might have been helpful. We will address these issues in a follow-up study for which we will perform power calculations based on our findings from this pilot study; however, we feel that we gained enough insight into patient preferences that enable the design of a methodologically sound follow-up study. Another limitation pertains to the limited interpretability of the findings due to discrepancies in the results. While most participants favored picture-personalized BPDs and reported increased motivation, this did not translate into significantly higher adherence. This suggests that visual personalization may enhance participant engagement but it does not necessarily lead to sustained behavioral change over time. Additionally, mood outcomes were inconsistent, with lower depression scores but slightly higher anxiety levels observed in the P‑BPD group. Although neither of these differences reached statistical significance, they underline the complex psychological effects of personalization. Lastly, we acknowledge that our study did not cover all aspects of BPD adherence, for example overreporting and underreporting of measured values [12].

In line with our intention to increase BPD adherence by fostering patient empowerment based on the inclusion of personalized images, we thoroughly explored how patients engaged with their BPDs. This strength of our study allowed us to evaluate factors affecting adherence other than inserted images (such as the presence of external reminders or previous experience with BPDs). Our open and real-world recruitment setting aiming to somewhat increase representability may be considered as another strength of our report.

In conclusion, one of the key findings of our pilot study was that the inclusion of personalized images to BPDs for people with stroke diagnoses is feasible. As BP management (e.g., through use of BPDs) remains a hallmark in secondary stroke prevention, any measure sustaining adherence over a long period is worth investigating. Our pilot study lays the ground for future research as we address relevant aspects of adherence to BP home monitoring and explored potential implications of patient preferences and perspectives. Lastly, our study enables robust sample size calculations and may facilitate the definition of other reasonable study endpoints in the future. These may include, besides BPD adherence, secondary adherence metrics such as precision of documentation, clinical impact of (P-)BPD use and psychological as well as behavioral outcomes such as patient engagement and mental health outcomes.

## Supplementary Information

Supplementary Table 1. Additional sociodemographic and clinical information of participants

## Data Availability

Data are available from the corresponding author upon reasonable request and after approval from the ethics review board at the Medical University of Vienna.
