# Supplementary material for: A randomized controlled pilot study investigating adherence to blood pressure diaries with personal pictures in stroke follow-up care
Source: Wien Klin Wochenschr. 2025 Apr 22;137(21-22):693–701. doi: 10.1007/s00508-025-02530-w (PMC12592310; doi:10.1007/s00508-025-02530-w)
Supplement: Supplementary file 1 — Supplementary Table 1. Additional sociodemographic and clinical information of participants [file 508_2025_2530_MOESM1_ESM.docx]

Supplementary Material

# Supplementary Table

**Supplementary Table 1.** **Additional sociodemographic and clinical information of participants.**

| **Parameter** | **Category** | **Total cohort**  **n = 19** | **R-BPD**  **n = 10** | **P-BPD**  **n = 9** |
| --- | --- | --- | --- | --- |
| **Occupation type (before hospitalization)**^a^ | mainly seated | 6 (32) | 3 (30) | 3 (33) |
|  | physically demanding | 1 (5.3) | 1 (10) | 0 (0) |
|  | mixed sitting/moving | 2 (11) | 0 (0) | 2 (22) |
|  | inability to work/retired | 10 (53) | 6 (60) | 4 (44) |
| **Employment status (before hospitalization)**^a^ | part-time | 2 (11) | 0 (0) | 2 (22) |
|  | full-time | 6 (32) | 3 (30) | 3 (33) |
|  | retired | 10 (53) | 6 (60) | 4 (44) |
|  | unemployed | 1 (5) | 1 (10) | 0 (0) |
|  | in training | 0 (0) | 0 (0) | 0 (0) |
| **Duration of**  **hospitalization**^a^ | days | 9 ± 22 | 18 ± 22 | 9 ± 8 |
| **Medication (at discharge)**^a^ | antidiabetics | 6 (32) | 4 (40) | 2 (22) |
|  | antidepressants | 5 (26) | 4 (40) | 1 (11) |
|  | analgetics | 2 (11) | 1 (10) | 1 (11) |
|  | antiplatelet therapy | 17 (90) | 10 (100) | 7 (78) |
|  | anticoagulant | 3 (16) | 2 (20) | 1 (11) |
|  | lipid-modifying drug | 15 (79) | 10 (100) | 5 (56) |
|  | other | 11 (58) | 6 (60) | 5 (56) |
| **Comorbidities**^a^ | diabetes | 5 (26) | 4 (40) | 1 (11) |
|  | prior stroke | 1 (5) | 1 (10) | 0 (0) |
|  | hyperlipidemia | 12 (63) | 9 (90) | 3 (33) |
|  | smoking | 5 (26) | 3 (30) | 2 (22) |
|  | atrial fibrillation | 0 (0) | 0 (0) | 0 (0) |
|  | renal insuffiency | 3 (16) | 3 (30) | 0 (0) |
|  | other | 11 (58) | 5 (50) | 6 (67) |
| **Subgroup ischemic strokes (n=15): Stroke etiology**^a^ | macroangiopathic | 8 (53) | 6 (67) | 2 (33) |
|  | small-vessel disease | 2 (13) | 2 (22) | 0 (0) |
|  | other | 1 (7) | 0 (0) | 1 (17) |
|  | undetermined | 4 (27) | 1 (11) | 3 (33) |

^a^absolute number (percentage); ^b^median ± interquartile range. Other (non-categorized) comorbidities included migraine, gastritis, spinal disc herniation, heart failure, diabetic retinopathy, psoriasis, breast cancer and hypothyroidism. Other (non-categorized) medications were oral contraceptives, proton pump inhibitors, eye droplets, vitamins, antiemetics, diuretics, insulin and levothyroxine. Other (non-categorized) types of initial stroke therapy were discontinuation of anticoagulants (in a case with hemorrhagic stroke) or no therapy. P-BPD = picture blood pressure diary; R-BPD = regular blood pressure diary
